# Using weight-for-age as a screening tool for metabolic syndrome in apparently healthy adolescents

**DOI:** 10.1038/s41390-024-03465-0

**Published:** 2024-08-12

**Authors:** Noa Oselka, Gal Dubnov-Raz, Tomer Ziv-Baran

**Affiliations:** 1 School of Medicine, Faculty of Medical and Health Sciences, Tel Aviv University, Tel Aviv, Israel; 2 Sheba Medical Center, Ramat-Gan, Israel; 3 School of Public Health, Faculty of Medical and Health Sciences, Tel Aviv University, Tel Aviv, Israel

## Abstract

**Background:**

The increasing prevalence of metabolic syndrome (MetS) among adolescents necessitates a simple and easy-to-use screening tool. This study aimed to develop and validate a simple model based on age, sex, race, and weight-for-age or BMI-for-age to identify adolescents with MetS.

**Methods:**

A cross-sectional study of adolescents (aged 12–18 years) who participated in the American National Health and Nutrition Examination Survey (NHANES) was performed. Participants with pre-existing hypertension, diabetes or dyslipidemia were excluded. Data from 2005–2018 were randomly divided into training (70%) and validation (30%) sets. Anthropometric, demographic data, and MetS criteria were extracted.

**Results:**

The training group included 1974 adolescents (52% boys, median age 15 years), and the validation group included 848 adolescents (50% boys, median age 14 years). Both weight- and BMI-for-age demonstrated good discrimination ability in the training group (AUC = 0.897 and 0.902, respectively), with no significant difference between them (*p* = 0.344). Multivariable models showed similar discrimination ability. Therefore, weight-for-age was chosen and using Youden’s index, the 93rd weight-for-age percentile (SDS 1.5) was identified as the optimal cut-off value for MetS. Similar values were observed in the validation group.

**Conclusions:**

Among adolescents aged 12–18 years, weight-for-age percentiles are an easy-to-use primary screening indicator for the presence of MetS.

**Impact:**

- The prevalence of metabolic syndrome in adolescents is increasing.
- An early detection screening tool is required to prevent related adulthood morbidity.
- Screening adolescents for metabolic syndrome is challenging.
- This study suggests the use of weight-for-age as a single criterion for primary screening of adolescents aged 12–18.
- Using weight-for-age as a single predictor of metabolic syndrome is expected to increase screening rates compared to using BMI-for-age, due to its simplicity.

## Introduction

In the last four decades, an increase in metabolic syndrome (MetS) rate was reported.^1–3^ In 2007, the International Diabetes Federation (IDF) developed criteria for defining MetS in children and adolescents.^4^ The criteria were inspired by the IDF guidelines for adults and other studies on the prevalence of MetS in children and adolescents.^5–10^ The IDF criteria require the presence of abdominal obesity plus the presence of at least two other components, (elevated triglycerides, low high-density lipoprotein (HDL) cholesterol, high blood pressure, and elevated plasma glucose).^4^ MetS in childhood is associated with an increased risk of developing cardiovascular disease in adulthood, along with other chronic diseases and even early death. MetS in childhood have odds ratios of 6.2 for tracking into adulthood, 2.3 to 11.5 for type 2 diabetes mellitus, 2.0 for elevated carotid artery thickness, and 14.6 for cardiovascular disease in adulthood.^11,12^

MetS is associated with increased healthcare burdens and costs.^13–15^ Therefore, early screening and primary prevention are essential. Usually, MetS is diagnosed at a community clinic visit by a professional staff. However, less than 50% of pediatricians and only 22% of all primary care physicians in the community measure anthropometric variables in children and youth.^16,17^ Moreover, the overall rate of lipid screening in community clinics among children and adolescents is low.^18,19^ and lipids are only measured in 27% of children with obesity in community clinics.^20^ Recently, a new easy-to-use MetS prediction model for self-evaluation among an apparently MetS-free adult population was introduced.^21^

Even though the importance of identifying MetS in youth to prevent adulthood chronic diseases is well published, to date, no easy-to-use self-evaluation screening tool is available. Therefore, to enable an easy-to-use Mets screening tool, this study aimed to develop and validate a simple model based on age, sex, race, and weight-for-age or BMI-for-age to predict the presence of MetS in apparently MetS-free individuals, aged 12–18 years.

## Methods

### Study design

The study is a cross-sectional study based on the U.S. National Survey of Health and Nutrition (NHANES) database. The NHANES is a continuous survey of the National Center for Health Statistics, which is part of the Centers for Disease Control and Prevention (CDC) with data released every two years.^22^ The NHANES assesses the health and nutrition status of children, adolescents, and adults in the U.S. population. The database is available online and used for a wide range of epidemiological studies.^7,10,21,23,24^ The NHANES samples are selected by a complex multidisciplinary probabilistic sample, representing the general U.S. population of all ages.

All participants aged 12–18 years from the 2005–2018 NHANES were included in the study. Participants who reported having major morbidities associated with MetS (i.e., diabetes, hypertension, lipid disorders) were excluded since the prediction model aims to screen for MetS in an apparently MetS-free population. In order to prevent a classification bias, participants with one or more missing criteria from the MetS definition were also excluded. Selection bias was evaluated by comparing those who were excluded according to these criteria with those who were included. All participants were randomly divided into training (70%) and validation (30%) groups. The training group was used to build the models and the validation group was used to verify the final model. The study was approved by the local Institutional Review Board (Approval number 5269-18-SMC).

### Variables

Several variables, as defined in the NHANES, were extracted to evaluate the association with metabolic syndrome. These variables can be reported by the individual or a family member without visiting a clinic and most of them have been studied previously^7,23,24^: age, sex, race (Caucasian/Hispanic/Black/multiracial), height, and weight. BMI was calculated as the individual’s weight in kilograms divided by the square of height in meters. Weight- and BMI-for-age standard deviation scores (SDS) as well as percentiles were calculated using the CDC growth charts.^25^

In addition, waist circumference, blood pressure measurements, and fasting blood tests (triglycerides, high-density lipoprotein (HDL) cholesterol, glucose), were extracted to evaluate the presence of MetS. Waist circumference (WC) Z-scores were calculated according to NHANES III.^26^ At the NHANES, three blood pressure measurements were taken for each participant. The mean of the three measurements was calculated for each participant and used to determine the criterion. The IDF criteria for MetS were used in the current study.^4^ According to these criteria, MetS diagnosis requires the presence of abdominal obesity plus the presence of at least two other components (elevated triglycerides, low HDL cholesterol, high blood pressure, and elevated plasma glucose). Abdominal obesity was defined as WC≥90^th^ percentile or adult cut-off if lower for ages <16 years or WC ≥ 94 cm for boys and ≥80 cm for girls for age 16+ years. Elevated triglycerides level was defined as triglycerides above 150 mg/dL (1.7 mmol/L). Low HDL cholesterol was defined as below 40 mg/dL (1.03 mmol/L) for ages <16 years and below 40 mg/dL (1.03 mmol/L) in boys and below 50 mg/dL (1.29 mmol/L) in girls for age 16+ years. High blood pressure was defined as systolic blood pressure equal to or greater than 130 mmHg or diastolic blood pressure equal to or greater than 85 mmHg. Elevated fasting plasma glucose was defined as greater than 100 mg/dL (5.6 mmol/L).

### Statistical analysis

The minimal sample size was calculated using a power of 80% and a significance level of 5%. Previous data showed that approximately 1 out of 20 individuals in the USA have MetS.^12^ Therefore, 1414 individuals were needed to identify variables with a low-medium association with MetS (effect size *d* = 0.35).

Categorical variables were summarized as frequency and percentage. The distribution of continuous variables was evaluated using a histogram and Q-Q plot and reported as mean and standard deviation (SD) or as median and interquartile range (IQR). The absolute standardized difference was used to evaluate the difference between the training and validation groups and between those who were included and excluded from the study. The standardized difference is a measure of the magnitude of the difference between two groups in standardized terms. It helps to compare the distribution of baseline covariates between two groups in observational studies. For continuous variables, the standardized difference is the difference in the mean of a variable between two groups divided by the standard deviation of the variable. It can also be used for comparing the prevalence of categorical variables between two groups. An absolute standardized difference of up to 10% (or 0.1) is considered a negligible difference between groups.^27^ The Chi-square test was used to compare categorical variables between the training and validation groups and the independent samples t-test and Mann-Whitney test were applied to compare continuous variables.

The NHANES has a complex probability sample and as such the statistical analysis requires incorporating sample design elements. Therefore, proper weighting was applied for both the univariate and multivariable analyzes.^28^ Univariate and multivariable logistic regression was used to evaluate the association between metabolic syndrome and each studied risk factor (age, sex, race, weight-for-age, and BMI-for-age). The regression models were used to estimate the probability of MetS in each participant in the training and validation groups and the area under the receiver operating characteristic (ROC) curve (AUC) was used to assess the discrimination ability of the model. The DeLong test was applied to compare the area under the receiver operating characteristic curves.^29^ To identify the optimal cut-off value, Youden’s J statistic was calculated for each potential cut-off value. The cut-off was set according to the maximal Youden’s J statistic. Sensitivities and specificities for cut-off values of 85th, 90th, and 95th percentiles as well as 1, 1.5, and 2 SDS were also reported. The positive likelihood ratio and negative likelihood ratio were presented for each cut-off value. Positive and negative predictive values were calculated based on previous reports of MetS prevalence.^12^ All statistical tests were two-sided. Statistical significance was defined as *p* < 0.05. SPSS software (IBM SPSS Statistics for Windows, version 28, Armonk, NY, 2021) and R: A Language and Environments for Statistical Computing (R Core Team, R Foundation for Statistical Computing, Vienna, Austria, 2023) were used for all statistical analyzes. The childsds package (Vogel M, childsds: Data and Methods Around Reference Values in Pediatrics, 2022) was used to calculate the WC-, weight- and BMI-for-age percentiles and SDS.

## Results

### Study population

During the study period, 70,190 individuals participated in the NHANES. Of them, 7686 were aged 12–18 years and 7231 were eligible for inclusion. A total of 4409 were excluded due to missing data of at least one MetS criteria. No demographic, BMI or weight differences were observed between those who met the inclusion criteria and those who did not (absolute standardized difference < 0.1, Appendix A). After exclusion, 2822 apparently MetS-free individuals aged 12–18 years were included in the study, 1947 in the training group, and 848 in the validation group (Appendix B). Participants’ characteristics are summarized in Table 1. MetS prevalence was similar in both groups (5.8% in the training group, and 5.1% in the validation group) as well as the other studied variables (absolute standardized difference < 0.1).

**Table 1:** Characteristics of individuals who participated in the NHANES and included in the study.

Characteristics	Training group	Validation group	Absolute standardized difference	*p*
Total number of participants	1974	848		
**Demographics**				
Age (years), median (IQR)	15(13–16)	14(13–16)	0.054	0.187
Male, *n* (%)	1028 (52.1)	425 (50.1)	0.040	0.340
Race, *n* (%)				0.134
Caucasian	548 (27.8)	249 (29.4)	0.035	
Hispanic	671 (34.0)	314 (37.0)	0.063	
Black	528 (26.7)	200 (23.6)	0.071	
Multiracial	227 (11.5)	85 (10.0)	0.048	
**Anthropometric**				
Weight				
Kilogram, median (IQR)	60.5 (51.2–72.8)	60.3 (49.8–72.8)	0.039	0.353
SDS, mean (SD)	0.81(1.13)	0.78 (1.17)	0.029	0.477
BMI				
Kilogram/Meter^2^, median (IQR)	22.1 (19.3–26.5)	21.8 (19.2–26.4)	0.037	0.402
SDS, mean (SD)	0.72 (1.11)	0.69 (1.12)	0.028	0.503
Waist circumference				
Centimeter, median (IQR)	76.9 (70.3–87.5)	76.4 (69.3–87.6)	0.042	0.250
SDS, mean (SD)	0.6 (0.92)	0.57 (0.92)	0.031	0.446
**Blood tests**				
Fasting glucose (mg/dL), median (IQR)	95 (89–99)	94 (90–99)	0.028	0.949
HDL-C (mg/dL), median (IQR)	52 (45–61)	52 (45–60)	0.002	0.770
Triglyceride (mg/dL), median (IQR)	65 (47–91)	66 (47–95)	0.057	0.499
**Blood pressure**				
Systolic (mmHg), median (IQR)	108 (102–115)	109 (102–115)	0.015	0.482
Diastolic (mmHg), median (IQR)	59 (52–66)	60 (52–67)	0.016	0.330
**MetS criteria**				
Waist circumference (WC), *n*(%)	654 (33.1)	280 (33.0)	0.002	0.954
Glucose, *n*(%)	470 (23.8)	199 (23.5)	0.007	0.844
Blood pressure, *n*(%)	47 (2.4)	16 (1.9)	0.035	0.415
HDL-C, *n*(%)	288 (14.6)	132 (15.6)	0.027	0.504
Triglycerides, *n*(%)	122 (6.2)	65 (7.7)	0.059	0.146
Has MetS by definition, *n*(%)	115 (5.8)	43 (5.1)	0.031	0.424

*BMI* body mass index, *HDL-C:* high-density lipoprotein cholesterol, *IQR:* interquartile range, *SD* standard deviation, *SDS* standard deviation score, *WC* waist circumference.

### Univariate and multivariable analysis of predictors of MetS

In the training group, male sex (OR = 2.44, 95% CI 1.42–4.20), Hispanic versus Caucasian ethnicity (OR = 2.47, 95% CI 1.36–4.48), Hispanic versus Black ethnicity (OR = 2.53, 95% CI 1.49–4.31), weight-for-age (SDS: OR = 6.91, 95% CI 5.14–9.29) and BMI-for-age (SDS: OR = 11.81, 95% CI 7.53–18.53) were associated with a higher probability of having MetS. Two multivariable models were performed. Both models included age, sex, and race, and either weight-for-age (SDS) or BMI-for-age (SDS). The univariate and multivariable analyses are summarized in Table 2.

**Table 2:** Weighted univariate and multivariable logistic regression models of potential predictors for the presence of metabolic syndrome.

	Univariate		Multivariable			
			Model 1		Model 2	
Characteristics	Weighted OR (95% CI)	*P*-value	Weighted aOR (95% CI)	*P*-value	Weighted aOR (95% CI)	*P*-value
**Demographics**						
Age (years)	1.02 (0.87–1.19)	0.820	1.17 (0.94–1.45)	0.157	1.07 (0.88–1.31)	0.497
Male	2.44 (1.42–4.20)	**0.001**	1.51 (0.84–2.72)	0.170	2.32 (1.27–4.24)	**0.007**
Race		**<0.001**		**<0.001**		**0.003**
Caucasian	1		1		1	
Hispanic	2.47 (1.36–4.48)		2.44 (1.16–2.09)		1.71 (0.84–3.49)	
Black	0.98 (0.49–1.93)		0.44 (0.20–0.96)		0.47 (0.22–0.99)	
Multiracial	0.86 (0.23–3.20)		0.91 (0.21–4.00)		0.88 (0.21–3.58)	
**Anthropometric**						
Weight-SDS	6.91 (5.14–9.29)	**<0.001**	7.48 (5.57–10.05)	**<0.001**		
BMI-SDS	11.81 (7.53–18.53)	**<0.001**			12.13 (7.61–19.35)	**<0.001**

Model 1: Age, Sex, Race, Weight-SDS.

Model 2: Age, Sex, Race, BMI-SDS.

*OR* odds ratio, *aOR* adjusted odds ratio, *BMI* body mass index, *SDS* standard deviation score.

Boldface indicates statistical significance (*p* < 0.05).

### Discrimination ability

**Fig. 1:**
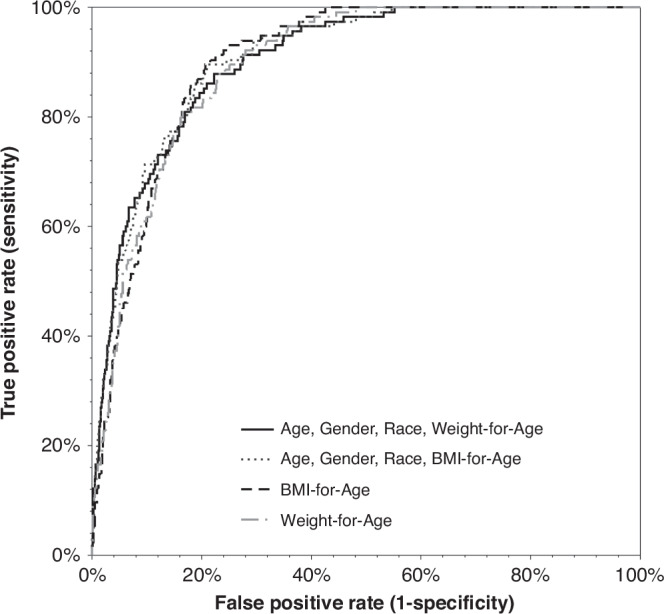
Receiver operating characteristic (ROC) curves demonstrating the discrimination ability of the models to predict MetS in the training group.

To evaluate the discrimination ability, the area under the ROC curve for weight-for-age, BMI-for-age, and the two multivariable models was calculated. The ROC curves and the areas under curves are summarized in Fig. 1 and Table 3. All models similarly showed a good discrimination ability (AUC ≈ 0.9) with no significant difference between them (*p* ≥ 0.150). Therefore, weight-for-age was chosen as the sole, easy-to-measure, preferred predictor for MetS.

**Table 3:** Discrimination ability of various weighted multivariable logistic regression models for the prediction of metabolic syndrome.

Model Parameters	AUC (95% CI)
Age, sex, race, weight-SDS	0.903 (0.880–0.927)
Age, sex, race, BMI-SDS	0.907 (0.884–0.929)
Weight-SDS	0.897 (0.875–0.918)
BMI-SDS	0.902 (0.882–0.922)

*BMI* body mass index, *SDS* standard deviation score.

**Fig. 2:**
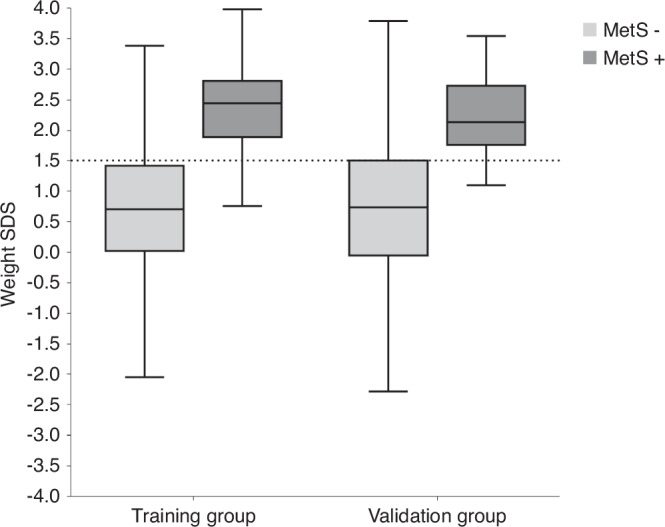
Box and whisker plot demonstrating the weight-for-age (SDS) among individuals who had MetS and who didn’t have across the study groups.

The optimal cut-off value of weight-for-age for predicting MetS was identified as 1.5 SDS which equals to 93.3 percentile. This cut-off value is illustrated for both, the training and validation groups in Fig. 2. In the training group, the optimal cut-off value presented a sensitivity of 87.8%, specificity of 76.9%, positive likelihood ratio of 3.82, and negative likelihood ratio of 0.16. The specificity and sensitivity values for additional cut-off values are presented in Table 4. The sensitivity and specificity while weighting were similar for each race (sensitivity 87–93%, specificity 70–80%, Appendix C). Using the optimal cut-off value and previously reported MetS prevalence in the US (4.5% to 8.4%),^12^ the positive predictive values and negative predictive values range between 15.2% and 25.9% and between 98.6% and 99.3%, respectively. The AUC for weight-for-age was similar in training (0.897, 95%CI 0.875–0.918) and validation groups (0.868, 95%CI 0.826–0.909, *p* = 0.225).

**Table 4:** Suggested weight-for-age cut-off values for the prediction metabolic syndrome.

Measure	Cut-off value	Sensitivity	Specificity	LR +	LR−
**Percentiles**	85.0%	96.5%	63.0%	2.608	0.056
	90.0%	92.2%	71.4%	3.224	0.109
	93.3%*	87.8%	76.9%	3.801	0.159
	95.0%	81.7%	80.3%	4.147	0.228
**SDS**	1.00	97.4%	61.7%	2.543	0.042
	1.50*	87.8%	76.9%	3.801	0.159
	2.00	68.7%	87.9%	5.678	0.356

*Identified as an optimal cut-off value using Youden’s index.

*LR* likelihood ratio, *SDS* standard deviation score.

## Discussion

MetS in childhood is associated with an increased risk of chronic diseases in adulthood.^11,12^ Studies employing the IDF adolescent criteria for MetS have reported prevalence rates ranging from 0.3% to 9.5%.^30^ In addition, high prevalence (40%) of undiagnosed MetS among adolescents with obesity and up to 14.3% undiagnosed prediabetes were reported.^31,32^ Therefore, early screening is essential for both secondary prevention of MetS and primary prevention of chronic diseases in adulthood.^33^ Screening for MetS is usually performed by primary care clinicians and involves anthropometric measurements, blood pressure assessment, and blood tests.^1,34–36^ Due to the low adherence to treatment during adolescence,^37^ routine screening for MetS is probably low. Recently, a new score for Mets screening in adolescents based on artificial intelligence (AI) methods was introduced. While the AI model demonstrated good discrimination ability, it still relied on anthropometric measurements and blood tests, which also require visiting the clinic.^38^ To the best of our knowledge, no easy-to-use self-evaluation screening tool is currently available. Therefore, this study aimed to develop and validate a model based on variables that can be easily reported by the individual or by a family member to identify MetS in apparently MetS-free adolescents.

This cross-sectional study included 1947 and 848 apparently MetS-free adolescents aged 12–18 years who participated in NHANES and served as the training group and validation group, respectively. The study showed that weight-for-age, as a sole predictor, has a good discrimination ability (AUC = 0.9), similar to that of BMI-for-age, and to that of models that also included age, sex, and race. Hence, by simply measuring body weight followed by a percentile calculation using an electronic application, the individual, family member or primary care physician can observe an increased risk for the presence of MetS and advocate further assessment.

Our findings may help to fill a gap highlighted by the technical report that served as a basis for the recent American Academy of Pediatrics clinical practice guidelines on obesity, stating that more information is needed on the specific amount of body fat or BMI levels at which metabolic aberrations occur and that there are currently too few data to determine whether youth with overweight or at the low end of obesity should be screened.^39^ By utilizing data from adolescents with a continuum of weight and BMIs, we were able to answer that question and identify a suitable cut-off (i.e., the 93rd weight-for-age percentile).

A previous study tried to predict Mets using anthropometric indices among Chinese adolescents.^40^ The study has reported that the BMI percentile can predict MetS with an AUC of 0.93, similar to that evaluated in the current study (AUC = 0.9). Another study that evaluated the performance of anthropometric indicators as predictors of MetS among Brazilian adolescents showed a lower discrimination ability (AUC of up to 0.73).^41^ In both studies, other anthropometric indicators also showed similar AUCs.^40,41^ BMI evaluation also requires height measurements, since during this life stage the height changes. Considering the low cooperation at this age, measuring height can make cooperation even more difficult. A cross-sectional survey of family physicians and pediatricians practicing in 4 US states (Alabama, Colorado, Massachusetts, and West Virginia) showed that only 44% of the physicians had strong intentions to measure BMI in pediatric and adolescent patients.^42^ Therefore, using weight measurement alone is expected to improve adherence to medical screening. While previous studies focus on BMI and other anthropometric measurements such as waist circumference and waist-to-height ratio, this study showed that weight can be an alternative measurement for BMI in MetS screening. Moreover, applying the optimal cut-off value that was identified (1.5 SDS), demonstrated only a small difference in the projected number of adolescents with MetS between BMI and weight in the screening for MetS (BMI: 271 out of 1000 individuals, weight: 268 out of 1000 individuals, of them 235 were identified by both metrics).

This study showed findings similar to those reported in previous studies that utilized the NHANES database in earlier periods (1988–1994, 1999–2008, 2001–2006).^7,23,24^ Age, as in the previous studies, was not associated with MetS, while male sex was associated with increased risk. MetS was also more prevalent in Hispanic adolescents than in Caucasian individuals and black adolescents had the lowest probability of having MetS. Consistent with the previous studies, higher BMI-for-age was found to be associated with a higher probability of having MetS.

The study suggests the use of weight-for-age as a single predictor for screening of MetS among adolescents aged 12–18 years. The discrimination ability of the weight-for-age was found to be high, both in the training group (AUC = 0.90) and the validation group (AUC = 0.87). A cut-off value of 1.5 SDS which equals to 93.3 percentile showed a sensitivity of 87.8%, specificity of 76.9%, positive likelihood ratio of 3.82, and negative likelihood ratio of 0.16. Therefore, weight-for-age has a good ability to discriminate between adolescents who do have MetS and those who do not and may be considered for primary self-evaluation screening. Moreover, these values were close in each race. To be practical and feasible for use in clinical practice, the 90th percentile can also be applied as a cut-off value with slightly higher sensitivity (92.2%) and lower specificity (71.4%). The 90th weight-for-age percentile was previously suggested to identify children and adolescents with obesity.^43^

Utilizing weight-for-age as the sole predictor for screening carries several implications for clinical practice. It facilitates straightforward screening in apparently MetS-free adolescents using an easily measurable parameter, which can be assessed by the individual or a family member without requiring visiting a clinic. Healthcare providers such as health maintenance organizations (HMO), can implement this approach to enhance early detection and precede a visit to the primary physician, by leveraging a mobile application or an online survey.

Since monitoring height in pediatrics is critical for various reasons, it is important to note that height measurement is critical during pediatric and adolescent clinic visits. A continuous effort is needed to increase the rate of height measurements in pediatric clinics.^44^

The study has several limitations. First, the use of self-reported or missing values for exclusion raises the possibility of a selection bias. To assess a selection bias, we compared excluded individuals to those included. Additionally, we used the NHANES database, a well-supervised survey database that randomly samples the US population and serves as a widely accepted data source for similar studies. In addition, we adhered strictly to the survey’s definitions. Second, the study’s external validity may be limited due to its focus on a US-only population. Cultural, socioeconomic, and healthcare system differences can influence the prevalence and risk factors of MetS in other countries, making it challenging to extrapolate the study’s conclusions to a global context. Therefore, further validation using data from other countries is warranted. However, the use of a large, nationally representative dataset like NHANES enhances the generalizability of the findings to a broader population. The sample weighting employed in this study helps ensure that the sample accurately represents the larger population from which it was drawn. Third, as a cross-sectional study, it was designed for secondary prevention of MetS and cannot identify factors for primary prevention. Fourth, pubertal status could also be included in the study as a potential predictor. A recent study evaluated data from NHANES III (1988–1994) showed that adjusting for pubertal status reduced the prevalence of overweight/obesity in the USA.^45^ However, pubertal status is not routinely incorporated in the NHANES dataset.

This study presents several notable strengths. First, the weight-for-age can be readily implemented for MetS screening across various platforms. Healthcare providers can utilize this approach by distributing a concise survey to adolescents and their parents to identify individuals with an increased risk of MetS. Second, while previous studies have relied on BMI-for-age, to our knowledge, this is the first model, that utilizes weight-for-age as a single predictor. Weight is a straightforward measurement, making it more likely that a larger proportion of adolescents will respond to the screening efforts. Third, the use of weight-for-age as a sole predictor for MetS screening was validated in a new group of adolescents, demonstrating similar predictive ability.

In conclusion, this study proposes a novel approach for MetS screening in adolescents. Utilizing weight-for-age as the sole predictor for MetS screening may enhance screening participation and, consequently, improve response rates. Beyond secondary prevention of MetS, this approach may help for primary prevention of chronic diseases in adulthood.

## Supplementary information

‏‏‏‏Appendixes

## Data Availability

The datasets analyzed during the current study are available on the U.S. National Center for Health Statistics website [https://wwwn.cdc.gov/nchs/nhanes/].
