# Supplementary material for: Using weight-for-age as a screening tool for metabolic syndrome in apparently healthy adolescents
Source: Pediatr Res. 2024 Aug 12;97(3):994–1000. doi: 10.1038/s41390-024-03465-0 (PMC12055599; doi:10.1038/s41390-024-03465-0)
Supplement: Supplementary file 1 — ‏‏‏‏Appendixes [file 41390_2024_3465_MOESM1_ESM.pdf]

Appendix A: Comparison of demographic and anthropometric data between those who were included and those who were excluded due to missing MetS criteria

**Characteristics**	**No missing MetS criteria**	**At least 1 missing MetS criteria**	**Absolute standardized difference**
Total number of participants	2,822	4,409	
**Demographics**			
Age (years), median (IQR)	14 (13-16)	14 (13-16)	0.000
Male, n (%)	1,453 (51.5)	2,253 (51.1)	0.008
Race, n (%)			
Caucasian	797 (28.2)	1,217 (27.6)	0.013
Hispanic	985 (34.9)	1450 (32.8)	0.043
Black	728 (25.8)	1,193 (27.1)	0.029
Multiracial	312 (11.1)	549 (12.5)	0.043
**Anthropometric**			
Weight			
Kilogram, median (IQR)	60.4 (50.9-72.8)	60.2 (51-73.1)	0.008
SDS, Mean (SD)	0.79 (1.141)	0.79 (1.151)	0.009
BMI			
Kilogram/Meter^2, median (IQR)	22 (19.2-26.5)	22.2 (19.5-26.3)	0.017
SDS, Mean (SD)	0.71 (1.11)	0.74 (1.101)	0.024

BMI - Body mass index; IQR - Interquartile range; SD - standard deviation; SDS - Standard deviation score

Appendix B: Flowchart of the selection of study groups from the U.S population that participated in the National Health and Nutrition Examination Surveys (NHANES), 2005-2018

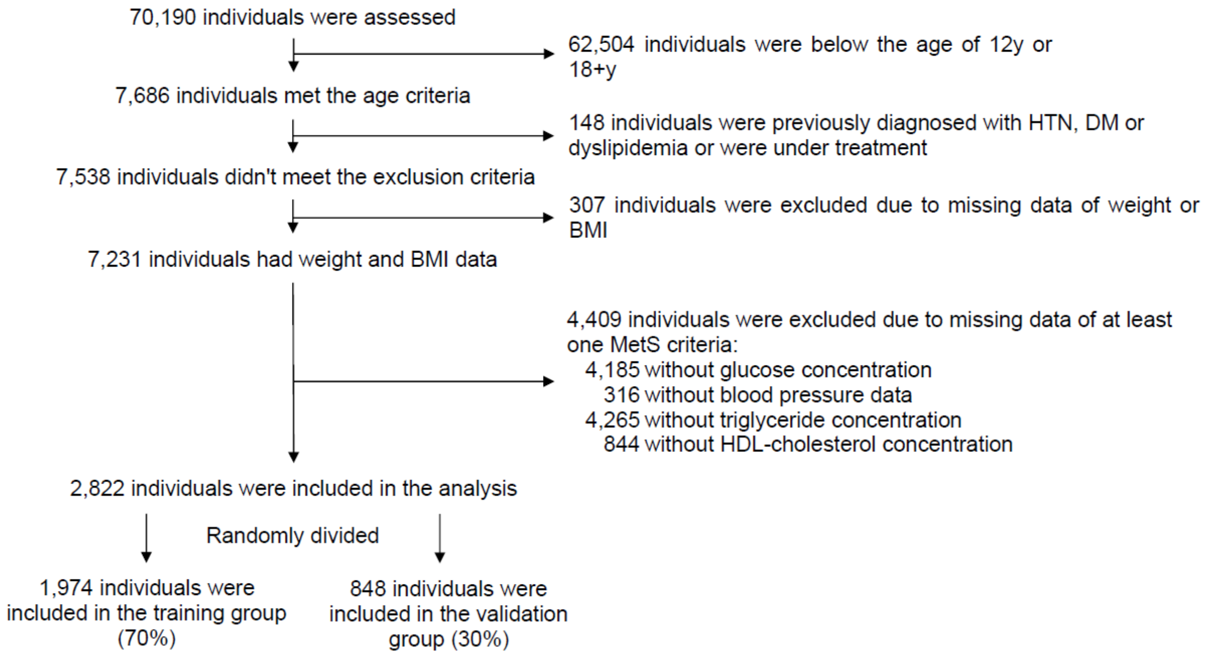

BMI - Body mass index; DM - Diabetes mellitus; HDL - High-density lipoprotein; HTN – Hypertension; WC - Waist circumference

Appendix C: Sensitivity and specificity values for the prediction of metabolic syndrome in each race using the identified optimal cut-off value (SDS = 1.5)

Race	Sensitivity	Specificity	LR+	LR-
**Caucasian**	92.3%	78.5%	4.293	0.098
**Hispanic**	86.7%	76.0%	3.613	0.175
**Black**	93.3%	70.1%	3.120	0.096
**Multiracial**	88.1%	80.1%	4.427	0.149
